# Mitochondrial Associated Ubiquitin Fold Modifier-1 Mediated Protein Conjugation in *Leishmania donovani*

**DOI:** 10.1371/journal.pone.0016156

**Published:** 2011-01-14

**Authors:** Sreenivas Gannavaram, Paresh Sharma, Robert C. Duncan, Poonam Salotra, Hira L. Nakhasi

**Affiliations:** 1 Laboratory of Emerging Pathogens, Division of Emerging and Transfusion Transmitted Diseases, Center for Biologics Evaluation and Research, U.S. Food and Drug Administration, Bethesda, Maryland, United States of America; 2 Institute of Pathology (ICMR), Safdarjung Hospital Campus, New Delhi, India; Louisiana State University, United States of America

## Abstract

In this report, we demonstrate the existence of the ubiquitin fold modifier-1 (Ufm1) and its conjugation pathway in trypanosomatid parasite *Leishmania donovani*. LdUfm1 is activated by E1-like enzyme LdUba5. LdUfc1 (E2) specifically interacted with LdUfm1 and LdUba5 to conjugate LdUfm1 to proteinaceous targets. Mass spectrometry analysis revealed that LdUfm1 is conjugated to *Leishmania* protein targets that are associated with mitochondria. Immunofluorescence experiments showed that *Leishmania* Ufm1, Uba5 and Ufc1 are associated with the mitochondria. The demonstration that all the components of this system as well as the substrates are associated with mitochondrion suggests it may have physiological roles not yet described in any other organism. Overexpression of a non-conjugatable form of LdUfm1 and an active site mutant of LdUba5 resulted in reduced survival of *Leishmania* in the macrophage. Since mitochondrial activities are developmentally regulated in the life cycle of trypanosomatids, Ufm1 mediated modifications of mitochondrial proteins may be important in such regulation. Thus, Ufm1 conjugation pathway in *Leishmania* could be explored as a potential drug target in the control of Leishmaniasis.

## Introduction

Leishmaniasis is a spectrum of diseases caused by protozoan parasites belonging to several different *Leishmania* species. These blood borne pathogens are currently prevalent in 88 countries around the World with an estimated 2 million new cases each year [1]. At present there are no effective vaccines against any of the clinical forms of leishmaniasis. Currently available therapeutic regimens are often limited in effectiveness due to unwarranted side effects and rapidly emerging drug resistance. Therefore, the quest for a novel vaccine and therapeutic targets acquires urgency towards controlling leishmaniases.

Gene expression regulation in eukaryotic cells occurs at various levels. In addition to initiation of transcription and post-transcriptional changes, a wide range of post-translational modifications are known to occur in eukaryotic cells. Collectively, these modifications greatly expand the functional diversity of the proteome. For this reason, protein modifications by ubiquitin and ubiquitin like proteins continue to be an intensely studied phenomenon [2]. The classical ubiquitin, a 8.6 kDa protein is conjugated to its substrate protein through a complex cascade of enzymatic reactions and signals targeting the protein to the proteasome for degradation [3]. Apart from ubiquitin, a growing list of small ubiquitin like proteins called Ubls is being discovered [4]. These Ubls possess essentially the same three dimensional structures as ubiquitin and employ mechanisms that generally follow the ubiquitin prototype for conjugation to protein substrates. These Ubls regulate a variety of biological functions ranging from endocytosis, membrane trafficking, protein kinase activation, DNA repair and chromatin dynamics [5], [6].

The diversity of functions regulated by the Ubls in eukaryotic organisms in general and the fact that inhibitors of the ubiquitin-proteasome pathway are either in clinical use [7] or are being studied for their potential as anticancer drugs [8], [9] suggests that it may be important to study these pathways in human parasitic organisms. Hence, systematic studies of Ubl pathways in the human trypanosomatid parasites such as *Leishmania* could yield better understanding of the pathogenesis on one hand and importantly could lead to novel therapeutic reagents. In *Leishmania*, most of gene expression regulation occurs post-transcriptionally [10]. Earlier studies on ubiquitin in trypanosomatid parasites such as *T. brucei* and *T. cruzi* focused on revealing the ubiquitin gene structure [11], [12], ubiquitin-dependent protein degradation [13], [14] and its role in differentiation from trypomastigote into an amastigote [15], [16]. Developmental regulation of polyubiquitin genes has been reported in *Plasmodium* [17]. Studies in *Plasmodium* identified deubiquitinating/deNeddylating activities [18] and sumoylation of telomere associated protein PfSir2, a novel substrate protein for SUMO [19]. Recent studies have demonstrated the role of ubiquitylation in the degradation of transmembrane surface proteins in trypanosomes [20], cell cycle regulation by the single SUMO homolog in *T. brucei* [21] and interactions with several nuclear proteins in the host cell by a protein that possesses an ubiquitin ligase activity secreted by *T. cruzi* [22]. However, even after completion of the genome sequencing of several of the trypanosomatid parasites, studies elucidating Ubl pathways, their conjugation and deconjugation mechanisms and the consequent modifications to the parasite proteome are absent. A recent review catalogued several of the Ubls and their deconjugating enzymes in parasitic protozoa including *Leishmania* and *Trypanosoma* [23].

Recently, a novel Ubl named ubiquitin-fold modifier 1 (Ufm1) that conjugates to target protein(s) has been identified in mammalian cells [24]. Ufm1 is synthesized as a precursor and a C-terminal cleavage reaction involving specific cysteine proteases, UfSP1 and UfSP2, exposes the conserved single glycine residue [25]. The mature Ufm1 thus generated is activated by a specific E1-like enzyme, Uba5, transferred to an E2- like enzyme, Ufc1, and ligated to the only substrate protein identified so far C20orf116, by a E3-like ligase Ufl1 [26]. However, the biological roles of the Ufm1-modification of cellular proteins remain unknown in part because of the difficulty in identification of the substrate proteins. In this report we demonstrate the presence of the Ufm1 pathway in the unicellular trypanosomatid parasite *Leishmania donovani*. We have identified the putative genes encoding the Ufm1, Uba5 and Ufc1 proteins and demonstrated the activities of *Leishmania* encoded Uba5 and Ufc1. We demonstrated for the first time the presence of Ufm1 modification pathway and the protein targets in the mitochondria. We also showed that modification/alteration of Ufm1 or Uba5 expression results in reduced survival of *Leishmania donovani* in infected human macrophages suggesting their role in *Leishmania* pathogenesis.

## Results

### Identification of genes encoding putative Ufm1 proteins in trypanosomatid genomes

Parasitic protozoan organisms including *Leishmania, Trypanosoma* and *Plasmodium* have complex life cycles and typically involve invertebrate and vertebrate hosts. As a consequence, several of the life-cycle transitions in these parasites must be accompanied by well regulated changes in their protein functions. Recent discoveries in the Ub and Ubl biology and the diversity of functions the Ubls regulate by means of protein modifications rekindled an interest in Ubl pathways in the parasitic organisms. Our previous studies analyzing life-cycle associated gene expression patterns in *Leishmania donovani* employing a genomic microarray revealed a gene fragment encoding a ∼43 kDa protein to be abundantly expressed in the amastigote stage of the parasite [27]. Bioinformatic analysis revealed that this *L. donovani* ∼43 kDa protein is homologous to human Uba5. Since human Uba5 has been shown to be the activating enzyme for Ufm1, we further searched trypanosomatid genomes and identified genes encoding putative Ufm1 proteins in the genome databases of *Leishmania infantum* (LinJ16_V31100) and *Trypanosoma brucei* (Tb927.8.5380). We PCR amplified a putative gene for Ufm1 from *L. donovani* genomic DNA. The predicted open reading frame of *L. donovani* Ufm1 encodes a ∼12.5 kDa protein that possesses a 17 aminoacid residue extension at the C-terminus where as the putative Ufm1 proteins in *T. brucei* and *T. cruzi* do not have this extension, similar to the human Ufm1 protein as seen by alignment of the trypanosomatid aminoacid sequences with the human sequence (Figure S1). A comparison of trypanosomatid Ufm1 aminoacid sequences with the human ortholog showed several blocks of conserved aminoacid residues including the C-terminal Gly which is important for conjugation to substrate proteins in several Ubls [4]. Overall, primary aminoacid sequence comparison revealed that *Leishmania* and *Trypanosoma* Ufm1 proteins display ∼60% identity with human Ufm1.

A comparison of the putative trypanosomatid Uba5 aminoacid sequences with the human ortholog showed several blocks of conserved aminoacid residues (Figure S2). Overall, primary aminoacid sequence comparison revealed that *Leishmania* and *Trypanosoma* Uba5 proteins display ∼50% identity with human Uba5. Further analysis of the *L. donovani* putative homolog of human Uba5 revealed it has an ATP-binding motif also common in other trypanosomatid parasites and is similar to human Uba5 and other E1-like enzymes (Figure S2). In addition, a metal binding motif shared by many E1-like enzymes such as human Uba5, Uba2, Uba3, Uba4 and Atg7 and an active site Cys residue occurring downstream of the metal binding motif was also found in trypanosomatid Uba5 (Figure S2). Together, sequence homology and conservation of catalytically important residues in the putative Ufm1 and also in Uba5 suggested that trypanosomatid genomes contain genes encoding some of the components of Ufm1 conjugation machinery. Therefore, we explored the biochemical characteristics of Ufm1 mediated protein modification machinery in *Leishmania*.

### LdUba5 interacts with LdUfm1 and displays E1 activity

**Figure 1:**
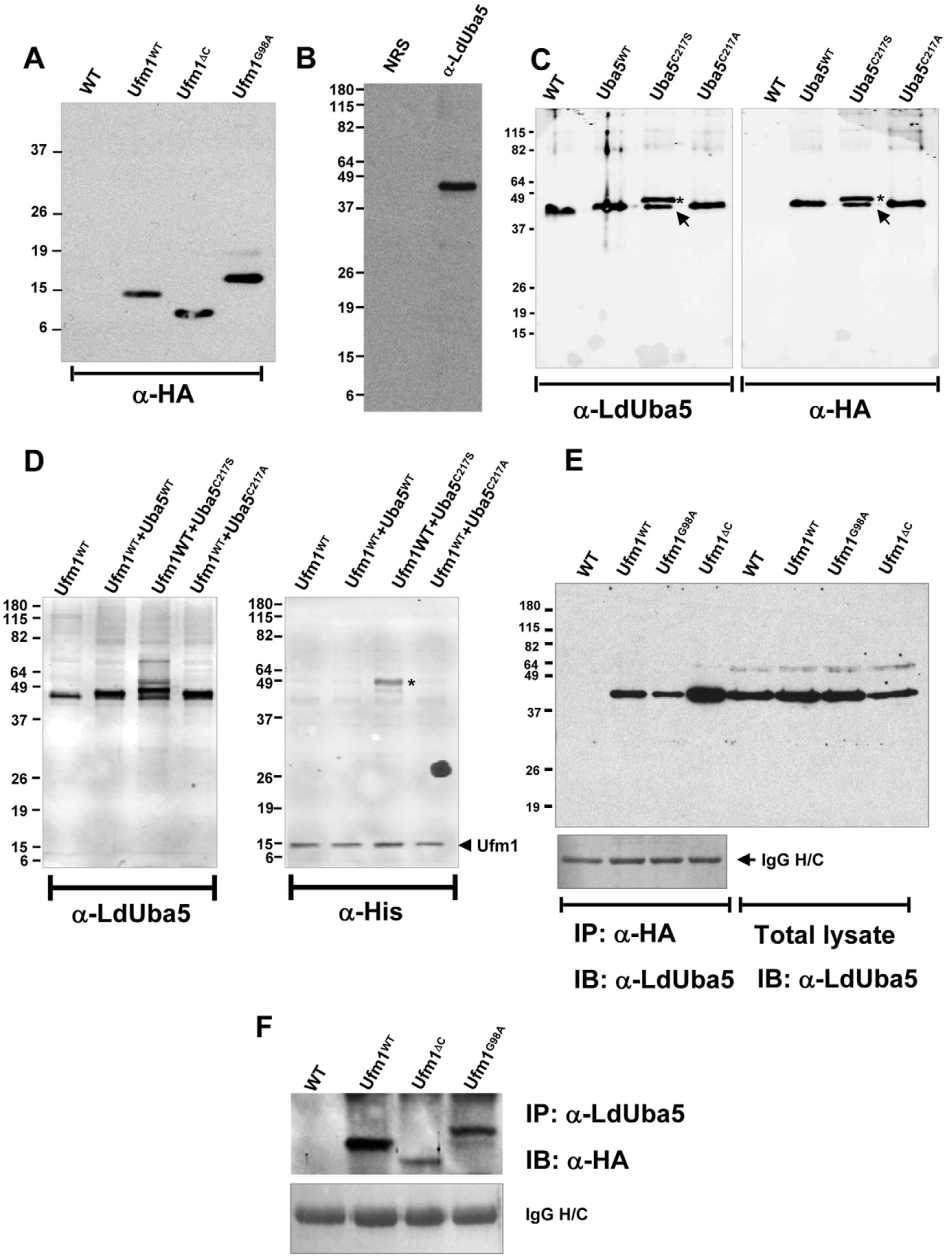
Protein-protein interactions between LdUba5 and LdUfm1. (A) Exogenous expression of Wild type LdUfm1 and the mutant forms. *Leishmania* transfectants overexpressing either wild type full length Ufm1 (Ufm1^WT^) or truncated form in which 18 aminoacid residues at the C-terminus including Gly^98^ were removed (Ufm1^ΔC^) or the mutant form in which the putative cleavage site Gly 98 is altered (Ufm1^G98A^) were prepared. The transfectants Ufm1^WT^, Ufm1^ΔC^ and Ufm1^G98A^ carried an HA epitope tag at the N-terminus. Protein lysates from equal number of the transfectants (Uba5^WT^, Uba5^C217S^ and Uba5^C217A^) and *L. donovani* wild type parasites (WT) were resolved on SDS-PAGE and probed with anti-HA antibodies (α-HA). (B) IgG fraction enriched from the polyclonal antibodies raised against *L. donovani* Uba5 protein were reacted with promastigote *L. donovani* lysates (α-LdUba5) in an immunoblot. Pre-immune serum was used as a control (NRS). (C) *Leishmania* transfectants overexpressing either wild type Uba5 (Uba5^WT^) or the mutant forms (Uba5^C217S^, Uba5^C217A^) in which the predicted active site Cys was changed to either Ser or Ala by PCR were prepared. The transfectants Uba5^WT^ and Uba5^C217A^ possessed an HA epitope tag at the C-terminus while in the Uba5^C217S^ mutant, the HA tag was at the N-terminus. Protein lysates from equal number of *L. donovani* wild type parasites (WT) and the transfectants (Uba5^WT^, Uba5^C217S^ and Uba5^C217A^) were resolved on SDS-PAGE and probed with either with polyclonal antibodies raised against LdUba5 (α-LdUba5) or with anti-HA antibodies (α-HA). (D) Identification of the stable intermediate linked to LdUba5 in *Leishmania* transfectant cells. *Leishmania* transfectants overexpressing Ufm1 containing both 6xHis and HA tags at the N-terminus (Ufm1^WT^) or co-expressing Ufm1^WT^ and either wild type Uba5 (Uba5^WT^) or the mutant forms (Uba5^C217S^, Uba5^C217A^) were prepared. Protein lysates from equal number of *L. donovani* transfectants expressing Ufm1^WT^ alone or co-expressing variants of Uba5 (Uba5^WT^, Uba5^C217S^ and Uba5^C217A^) were resolved on SDS-PAGE and probed with either with polyclonal antibodies raised against LdUba5 (α-LdUba5) or with anti-His antibodies (α-His). (E) Immunoblot analysis following immunoprecipitation reactions. Protein lysates from wild type *L. donovani* cells (WT) or transfectants expressing variants of LdUfm1 (Ufm1^WT^, Ufm1^G98A^ or Ufm1^ΔC^) were used in co-immunoprecipitation reactions with α-HA affinity gel and the eluates were resolved on SDS-PAGE and the immunoblots were probed with anti-LdUba5 antibodies (α-LdUba5). Ponceau-S stained immunoblot showing the heavy chain IgG (IgG H/C) to indicate the amount of antibodies used in immunoprecipitation reactions. The immunoblot of the total lysates with α-LdUba5 antibodies indicating equal amounts of lysates used in immunoprecipitation reaction is shown. (F) Conversely, protein lysates from wild type *L. donovani* cells (WT) or transfectants (Ufm1^WT^, Ufm1^G98A^ or Ufm1^ΔC^) were used in co-immunoprecipitation reactions with α-LdUBA5 antibodies and the immunoblots were probed with anti-HA antibodies (α-HA). Ponceau-S stained immunoblot showing the heavy chain IgG (IgG H/C) to indicate the amount of antibodies used in immunoprecipitation reactions.

To investigate if the *Leishmania* Ufm1 undergoes enzymatic processing prior to the activation by Uba5 as demonstrated in human Ufm1 [24], *Leishmania* transfectants were prepared that overexpress either the full length LdUfm1 (Ufm1^WT^) or a mutant in which the C-terminal Gly is changed to Ala (Ufm1^G98A^) or a variant of LdUfm1 in which 18 aminoacid residues at the C-terminus were removed including Gly 98 and therefore will not undergo processing (Ufm1^ΔC^). These transfectants carried an N-terminal HA-epitope tag. Immunoblotting with total lysates from these transfectants indicated that the exogenously expressed wild type Ufm1 indeed undergoes processing as revealed by its faster migration on the SDS-PAGE gel compared to Ufm1^G98A^ (Figure 1A) as was observed in the analysis of human Ufm1 [24]. The Ufm1^ΔC^ showed the fastest migration as can be expected and the wild type endogenously expressed Ufm1 (WT) did not show any reactivity with α-HA antibodies (Figure 1A). These results indicated that LdUfm1 undergoes enzymatic processing prior to activation and suggested the existence of an Ufm1 processing C-hydrolase activity in *L. donovani*.

It has been shown in many of the E1 and E1-like enzymes cysteine residue in the active site is important for its activation and interaction with the corresponding Ubl. Also, it has been demonstrated that changing the cysteine residue in the active site to serine results in formation of stable intermediates between the E1 and its respective modifier protein [24]. This stable intermediate is resistant to reducing agents and would be detected as a high molecular weight product on an immunoblot. To determine if the putative LdUba5 indeed can interact with LdUfm1 and activate it, *Leishmania* transfectants that overexpress either wild type Uba5 or mutants in the putative catalytic site (Cys217Ser or Cys217Ala) were prepared. The Cys217Ala Uba5 mutant was developed to serve a control for the Cys217Ser mutant. The wild type Uba5 and Uba5^C217A^ mutant carried a C-terminal HA-epitope tag where as the Uba5^C217S^ mutant carried an N-terminal HA-epitope tag. To identify the endogenous LdUba5, polyclonal antibodies were raised against the recombinant LdUba5 protein. These antibodies identified a distinct ∼43 kDa band in the *L. donovani* lysates (Figure 1B). Further, our results showed that in immunoblots probed with anti-LdUba5 antibodies, a high molecular weight intermediate was detectable only in cells overexpressing the LdUba5^C217S^ mutant (Figure 1C, indicated with *). These cells also express the endogenous wild type LdUba5 migrating as the lower molecular weight band (Figure 1C, indicated by arrowhead). In the cells expressing LdUba5^C217A^ or wild type Uba5^WT^ the high molecular weight intermediate was not found (Figure 1C). An anti-HA immunoblot of an identical panel of lysates showed the expression level of exogenously expressed LdUba5 variants to be comparable (Figure 1C). The high molecular weight intermediate observed in the LdUba5^C217S^ mutant with anti-LdUba5 antibodies was also evident in the anti-HA immunoblot (Figure 1C). These results suggest the conservation in the mechanism of LdUba5 similar to that found in other Ubl activating enzymes.

To verify that LdUfm1 is the endogenous protein with which LdUba5 forms a high molecular weight intermediate in the C217S mutant, *Leishmania* transfectants that co-express LdUfm1 in either wild type LdUba5 or LdUba5^C217S^ or LdUba5^C217A^ mutant background were prepared. The LdUfm1 overexpression construct carried an HA-epitope and 6xHis tags at the N-terminus. The lysates from these transfectants were first probed with α-LdUba5 antibodies and the immunoblot (Figure 1D) showed that these co-expressors showed a similar expression pattern to single expressors as shown in Fig. 1C. When the immunoblots were probed with an α-His antibody which will recognize only LdUfm1, a ∼60 kDa band was apparent only in the transfectant co-expressing LdUfm1 and LdUba5^C217S^ (Figure 1D α-His, indicated with *). This result demonstrated that in the LdUba5^C217S^ mutant, LdUfm1 co-expressed protein is part of the high molecular weight intermediate (Figure 1D). To further confirm this interaction between LdUba5 and LdUfm1, we expressed wild type and mutant forms of LdUfm1 and performed coimmunoprecipitation experiments (Figure 1E). Protein lysates from *Leishmania* transfectants expressing either wild type or mutant forms of Ufm1 (Ufm1^G98A^, Ufm1^ΔC^) containing HA-epitope tag were incubated with anti-HA affinity gel and the immunoprecipitated material was resolved on SDS-PAGE and the immunoblots were probed with anti-LdUba5 antibodies. The results showed that LdUba5 protein can be precipitated with all three forms of LdUfm1 indicated by the specific ∼43 kDa band in all the transfectants except in untransfected *Leishmania* cells that do not express Ha-tagged Ufm1 (Figure 1E) even though such molecular interactions are known to be transient in other Ubls. Conversely, when protein lysates from these cells expressing either wild type or mutant forms of Ufm1 (Ufm1^G98A^, Ufm1^ΔC^) were immunoprecipitated with anti-LdUba5 antibodies and the immunoblots from the immunoprecipitated material were probed with anti-HA that recognizes LdUfm1 protein or its mutant forms, 10–16 kDa bands representing the size of Ufm1 proteins were observed (Figure 1F) as was seen previously (Figure 1A).

**Figure 2:**
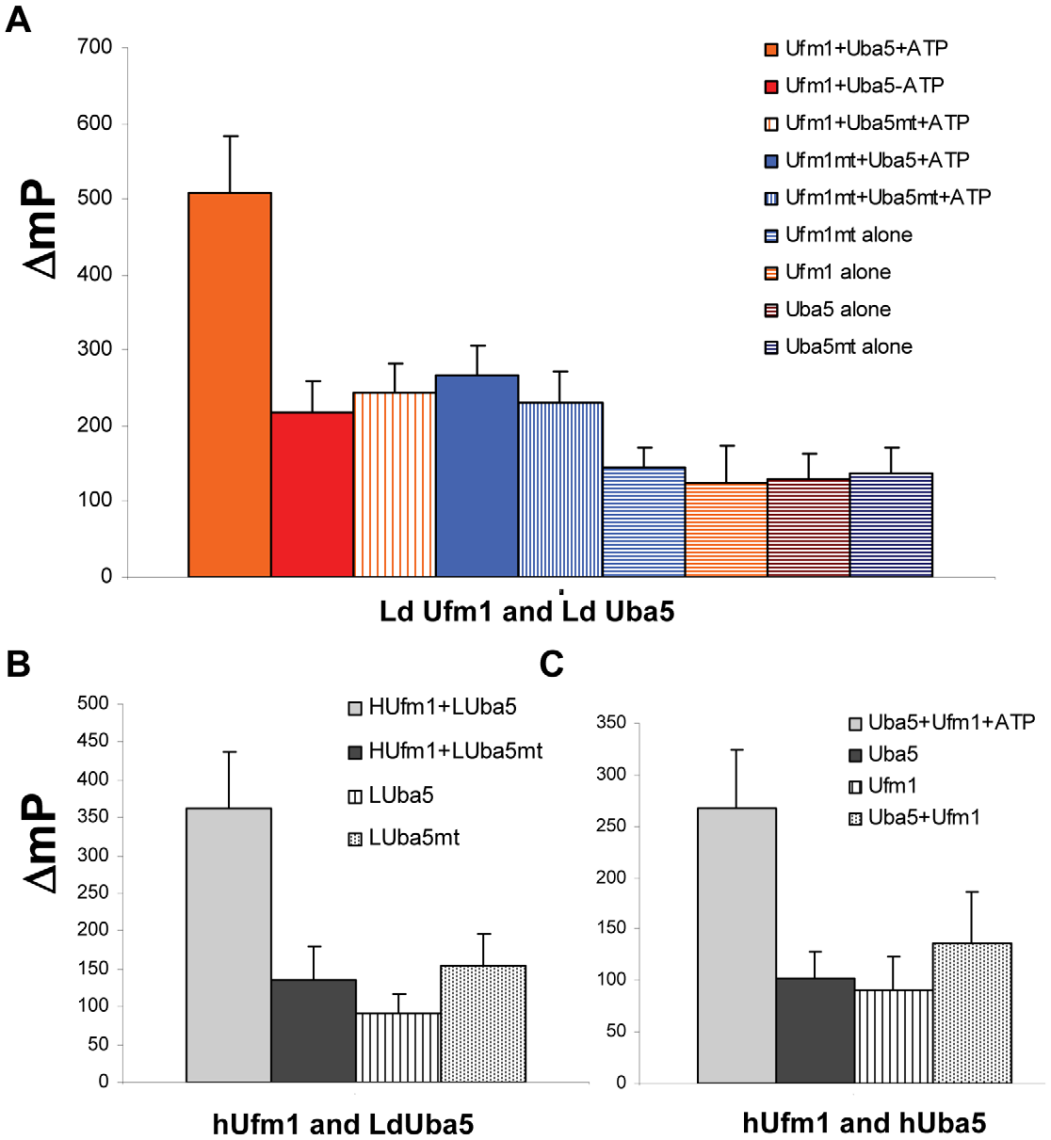
Demonstration of E1-like activity of LdUba5 and activation of LdUfm1. (A) Activation of *Leishmania* Ufm1 by LdUba5. *Leishmania* Ufm1 lacking the 17 aminoacid residues at the C-terminus and thus exposing the terminal Gly residue and a mutant Ufm1 in which the terminal Gly is changed to Ala were immunoprecipitated from the respective *Leishmania* transfectants were incubated with either wild type LdUba5 or LdUba5^C217A^ proteins immunoprecipitated from the corresponding *Leishmania* transfectants in the combinations indicated either with or without ATP and changes in the magnitude of fluorescent polarization are measured. *Leishmania* proteins from LdUba5, LdUba5^C217A^, LdUfm1 or LdUfm1^G98A^ transfectants were included as background controls. The data are collected from three independent experiments. Error bars indicate the standard deviation. (B) Activation of human Ufm1 by LdUba5. Human Ufm1 was incubated with either wild type LdUba5 or LdUba5^C217A^ proteins immunoprecipitated from the corresponding *Leishmania* transfectants and ATP and changes in the magnitude of fluorescent polarization are measured. *Leishmania* proteins from LdUba5 or LdUba5^C217A^ transfectants without human Ufm1 were included as background controls. (C) Human recombinant Uba5 and Ufm1 proteins were incubated either in the presence or absence of ATP and changes in the magnitude of fluorescent polarization in the tracer molecule are measured indicating the activity of Uba5.

To verify whether LdUba5 not only interacts with LdUfm1 but possesses E1-like activity, an end point fluorescence polarization assay was utilized. We tested wild type *Leishmania* Uba5 and LdUba5^C217A^ proteins for the E1-like activity using either wild type LdUfm1 or the mutant LdUfm1^G98A^ as substrates. The wild type LdUfm1 used in this assay contained Gly98 as the C-terminal residue and therefore would appear as mature LdUfm1. Similar to LdUba5 proteins, HA-epitope tag containing LdUfm1 variants were also immunoprecipitated from corresponding *Leishmania* transfectants using an α-HA affinity gel. The results showed that E-1 like activity was evident only when wild type LdUba5 and wild type mature LdUfm1 along with ATP were present in the reaction and in the presence of either LdUba5 or LdUfm1 mutants such activity was not detected (Figure 2A). On the other hand, in the absence of ATP, there was no detectable E-1 like activity with wild type LdUba5 and wild type mature LdUfm1 (Figure 2A). To test whether LdUba5 can activate mature human Ufm1 and thus functionally complement human Uba5, we used recombinant mature human Ufm1 with either wild type LdUba5 or LdUba5^C217A^ proteins immunoprecipitated from the respective *Leishmania* transfectants with α-HA affinity gel. Results showed that wild type LdUba5 can activate human Ufm1 as measured by the decreased fluorescence polarization observed only in the wild type LdUba5 but absent in the LdUba5^C217A^ protein (Figure 2B). In control experiments, we tested human Uba5 and human Ufm1 in a similar activation assay. Results showed that human recombinant Uba5 displays E-1 like activity only when all the components i.e., Uba5, Ufm1 and ATP are present (Figure 2C). Together, these results clearly established the E-1 like activity of LdUba5 and activation of LdUfm1. Furthermore, LdUba5 can complement human Uba5 in an *in vitro* assay for E1 like activity.

### LdUfc1 interacts with LdUfm1

**Figure 3:**
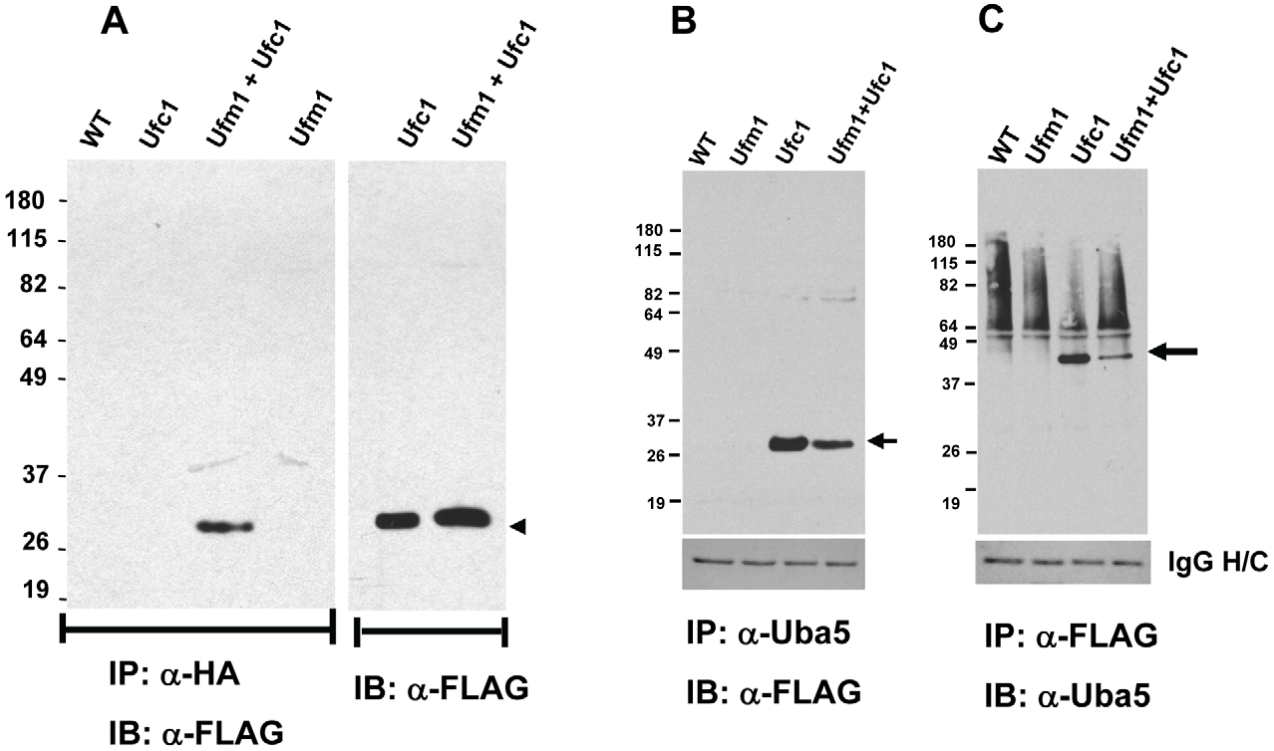
Expression of LdUfc1, the conjugating enzyme of Ufm1 in *Leishmania* and protein interactions between LdUfc1, LdUba5 and LdUfm1. (A) Immunoblot analysis following immunoprecipitation reactions. Protein lysates from wild type *L. donovani* cells (WT) or transfectants expressing either the putative LdUfc1 with a FLAG epitope or LdUfm1 or coexpressing the putative LdUfc1 and LdUfm1 were used in co-immunoprecipitation reactions with α-HA affinity gel and the eluates were resolved on SDS-PAGE and the immunoblots were probed with anti-FLAG antibodies. The immunoblot of the total lysates with α-FLAG antibodies indicating equal amounts of lysates used in immunoprecipitation reaction is shown (arrow head). (B) Immunoblot analysis following immunoprecipitation reactions. Protein lysates from wild type *L. donovani* cells (WT) or transfectants expressing either the putative LdUfc1-FLAG or LdUfm1 or coexpressing the putative LdUfc1-FLAG and LdUfm1 were used in co-immunoprecipitation reactions with α-LdUba5 antibodies and the eluates were resolved on SDS-PAGE and the immunoblots were probed with anti-FLAG antibodies. The bands corresponding to LdUfc1 are indicated with arrow mark. Ponceau-S stained immunoblot showing the heavy chain IgG (IgG H/C) to indicate the amount of antibodies used in immunoprecipitation reactions. (C) Conversely, protein lysates from wild type *L. donovani* cells (WT) or transfectants expressing either the putative LdUfc1-FLAG or LdUfm1 or coexpressing the putative LdUfc1-FLAG and LdUfm1 were used in co-immunoprecipitation reactions with α-FLAG antibodies and the eluates were resolved on SDS-PAGE and the immunoblots were probed with anti-LdUba5 antibodies. The bands corresponding to LdUba5 are indicated with arrow mark. Ponceau-S stained immunoblot showing the heavy chain IgG (IgG H/C) to indicate the amount of antibodies used in immunoprecipitation reactions.

Existence of a functional LdUfm1and its activating enzyme LdUba5 that has E1-like activity indicated that other components such as conjugating enzymes might also be present in the trypanosomatid parasites. Bioinformatic analysis using human Ufc1 protein that has been previously shown to be the conjugating enzyme of human Ufm1 [24] allowed us to identify genes encoding putative Ufc1 proteins in the genomes of *Leishmania infantum* (LinJ15_V3.1270), *Trypanosoma brucei* (Tb09.160.4150) and *Trypanosoma cruzi* (Tc00.1047053506445.100). A comparison of the putative trypanosomatid Ufc1 aminoacid sequences with the human ortholog showed several blocks of conserved aminoacid residues including the Cys115 residue which has been shown to be the active site in human Ufc1 (Figure S3 indicated by *). Overall, primary aminoacid sequence comparison revealed that *Leishmania* and *Trypanosoma* Ufc1 proteins display ∼60% identify with human Ufc1. To ascertain if the putative LdUfc1 can interact with LdUfm1 for conjugation to potential protein targets, the putative Ufc1 coding sequence was amplified from the *L. donovani* genome and cloned into an expression vector. *Leishmania* transfectants that overexpress full length LdUfc1 alone or coexpress LdUfc1 and LdUfm1 proteins were prepared. The full length LdUfc1 carried a C-terminal FLAG-epitope tag where as the LdUfm1 carried an N-terminal HA-epitope tag. To detect the molecular interaction between these proteins, we performed coimmunoprecipitation experiments. Immunoprecipitation reactions followed by immunoblotting using lysates from the transfectants showed that the putative LdUfc1 interacts with LdUfm1 as revealed by the presence of the nearly ∼26 kDa LdUfc1 protein only in the transfectants that express both LdUfm1 and LdUfc1 and not when either of the epitope tagged proteins is expressed alone (Figure 3A, indicated by arrow). This result showed that the LdUfc1 can interact with LdUfm1 and potentially carry out the conjugation reaction. To test whether a molecular interaction could be detected between the endogenous LdUba5 and transfected LdUfc1, similar to human Uba5 and Ufc1, lysates from transfectants expressing LdUfm1, LdUfc1 alone or expressing both proteins were used in a coimmunoprecipitation reaction using α-LdUba5 antibodies followed by immunoblotting with α-FLAG antibody which recognizes LdUfc1. The result showed that FLAG tagged LdUfc1 can be detected in an immunoblot both in LdUfc1 transfectants and those expressing the combination of LdUfc1 and LdUfm1 (Figure 3B, indicated by arrow) but not in the untransfected *L. donovani* lysates (Figure 3B). On the other hand, when α-FLAG antibodies were used in the immunoprecipitation reaction, LdUfc1 could pull down endogenous LdUba5 protein as shown by the immuno-reactive ∼43 kDa band on the immunoblot using LdUba5 antibodies (Figure 3C, indicated by arrow). Together, these results demonstrate that LdUba5 and LdUfc1 can independently interact with LdUfm1 and also can be found in a molecular complex with all three components.

### Role of Ufm1 and Uba5 in the parasite pathogenesis

**Figure 4:**
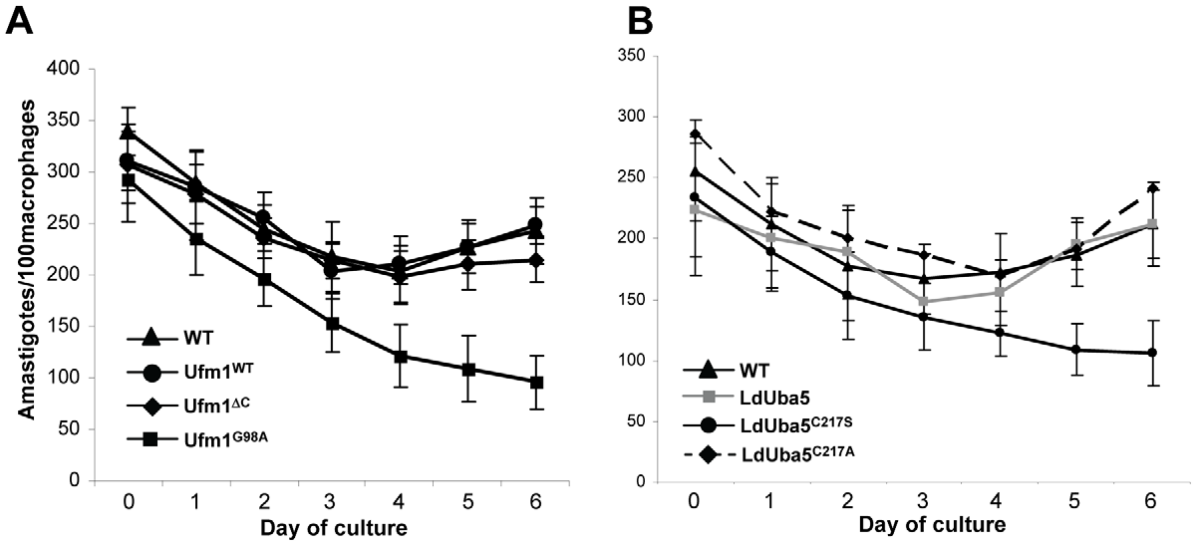
In vivo effects of alterations in the LdUfm1/Uba5 expression on *Leishmania* growth. (A) Human macrophages differentiated from monocytes were infected with purified metacyclic parasites from *L. donovani* wild type (WT) or transfectant cultures (Ufm1, Ufm1^G98A^ or Ufm1^ΔC^) for six hours (10∶1 parasite-to-macrophage ratio) and the numbers of amastigotes in these cultures were determined over a period of 6 days by microscopic observation of Diff-quik reagent stained slides. The data are expressed as the number of amastigotes per 100 macrophages. Error bars indicate the standard deviation. (B) The growth of *L. donovani* wild-type (WT), or wild-type Uba5 (LdUba5) or mutant Uba5 transfectants (LdUba5^C217S^ or LdUba5^C217A^) amastigotes in human macrophages was monitored. The results are the mean of three independent experiments.

To further investigate the role of LdUfm1 conjugation on the differentiation and survival of intracellular amastigotes, human macrophages derived from elutriated monocytes were infected with wild type stationary phase *Leishmania* promastigotes or those overexpressing either wild-type LdUfm1 or variants of LdUfm1 (Ufm1^G98A^, Ufm1^ΔC^) according to previously published protocol [28]. No difference in the number of amastigotes per hundred macrophages was found between wild type control cells or transfectants expressing either wild type Ufm1 or Ufm1^ΔC^ except parasites overexpressing the LdUfm1^G98A^ mutant. Over-expression of LdUfm1^G98A^ resulted in reduction in number of *Leishmania* amastigotes in the macrophages after 72 hours of infection. This reduction in parasite number is significant compared to control cells (WT) and LdUfm1^WT^ and LdUfm1^ΔC^ transfectants (*p*<0.005, Figure 4A). The reduction in amastigote number in LdUfm1^G98A^ mutants suggests that over-expression of non-processed Ufm1 probably disrupts the regulation of Ufm1 conjugation and also competes with the wild type Ufm1 for conjugation/deconjugation machinery and thereby results in dominant negative effects. To verify such disruption in regulation we performed Western blot analysis of proteins conjugated with Ufm1 and its mutant forms (Figure S4). We observed the loss of a subset of conjugated proteins with Ufm1^G98A^ mutant and complete loss of conjugating proteins with Ufm1^ΔC^ (Figure S4 compare lanes 2 and 3 with lane 1). It is therefore possible that the G98A mutation confers resistance against Ufm1 processing as shown in the figure 1A, and the protein conjugates formed with Ufm1^G98A^ may remain stable that could interfere with the function of the wild type Ufm1 and hence may affect the growth of parasites as observed by the reduction of parasite number in macrophages (Figure 4A). On the other hand, since we did not observe conjugating proteins with Ufm1^ΔC^, it is possible that it may not interfere with the function of wild type Ufm1 as was observed by the growth of parasites in macrophages (Figure 4A). The interaction between LdUfm1 and LdUba5^C217S^ mutant results in an irreversible complex formation and presumably resulting in reduced Ufm1 availability (Figure 1D). Therefore, we investigated the effects of active site mutations in the Uba5, the Ufm1 activating enzyme, on parasite growth in human macrophage infection experiments. A significant reduction in the growth of LdUba5^C217S^ mutant was observed in the macrophages compared with either the wild type or transfectants overexpressing wild type Uba5 or LdUba5^C217A^ mutant (Figure 4B). Together, these results demonstrated that alterations in LdUfm1 conjugation or indirectly mutations in LdUba5 affect the parasitic growth in the human macrophages.

### Mitochondrial localization of LdUfm1, LdUba5 and LdUfc1

**Figure 5:**
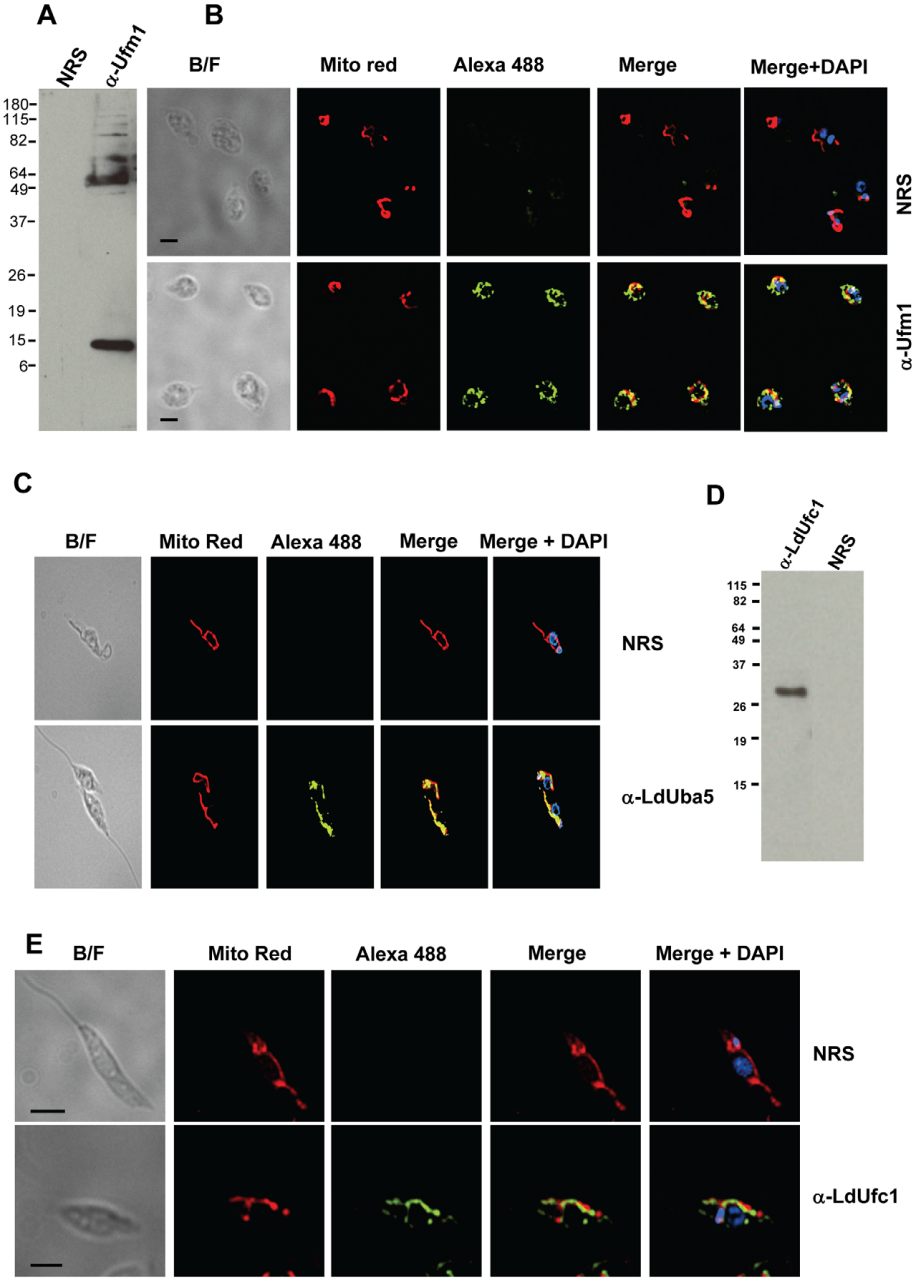
Immunofluorescence staining of *L. donovani* parasites with antibodies against LdUfm1, LdUba5 and LdUfc1. (A) Affinity purified polyclonal antibodies raised against *T. brucei* Ufm1 protein reacted with promastigote *L. donovani* lysates (α-Ufm1) in an immunoblot. Only a shorter exposure of the immunoblot is shown to avoid revealing the multiple faint bands resulting from Ufm1 conjugates. Pre-immune serum was used as a control (NRS). (B) *L. donovani* promastigote cells were stained with antibodies against TbUfm1 (α-Ufm1) as primary, and Alexa488-conjugated anti-rabbit IgG as secondary antibodies. To visualize mitochondria, cells were labeled with Mitotracker Red (Mito Red). The nucleus and kinetoplast were stained with DAPI. Pre-immune serum (NRS) was used in control staining experiments. (C) *L. donovani* promastigote cells were stained with IgG fraction enriched from anti-LdUba5 antiserum (α-LdUba5) as primary, and Alexa488-conjugated anti-rabbit IgG as secondary antibodies. Mitochondria, nucleus and kinetoplast were stained as described above. Pre-immune serum (NRS) was used in control staining experiments. (D) Affinity purified polyclonal antibodies raised against *L. donovani* Ufc1 protein were reacted with promastigote *L. donovani* lysates (α-LdUfc1) in an immunoblot. Pre-immune serum was used as a control (NRS). (E) *L. donovani* promastigote cells were stained with antibodies against LdUfc1 (α-LdUfc1) as primary, and Alexa488-conjugated anti-rabbit IgG as secondary antibodies. Mitochondria, nucleus and kinetoplast were stained as described above. Pre-immune serum (NRS) was used in control staining experiments. Bars, 5 µm (B, C) 10 µm (E).

The observation that all the LdUfm1 substrate proteins are localized in the mitochondria led us to investigate the cellular distribution of endogenous LdUfm1, LdUba5 and LdUfc1 proteins in *Leishmania.* To this end, antibodies against *L. donovani* LdUba5 and LdUfc1 and *T. brucei* Ufm1 proteins were used in immunofluorescence studies. There is a high degree of similarity between LdUfm1 protein and its *T. brucei* homolog resulting in cross reaction of LdUfm1 with the anti-TbUfm1 antibodies. An immunoblot with affinity-purified anti-TbUfm1 antibodies specifically recognized the unconjugated Ufm1 migrating at about ∼12 kDa and in addition a ∼50 kDa band, presumably an LdUfm1 conjugate in *L. donovani* promastigote lysates (Figure 5A). Immunofluorescence assays with these antibodies revealed that endogenous LdUfm1 is localized in mitochondria in *L. donovani* promastigotes, as indicated by its colocalization with Mitotracker Red, a mitochondrial marker (Figure 5B, bottom panel). No background reactivity was obtained with the pre-immune serum in these experiments (Figure 5B, top panel). The pattern of colocalization is likely to represent both the free pool of LdUfm1 and also the Ufm1 conjugated to the substrate proteins. We next explored the localization of other components of the Ufm1 machinery i.e., LdUba5 and LdUfc1. Immunofluorescence assays with the anti-LdUba5 antibodies revealed that endogenous *Leishmania* Uba5 in promastigotes is also clearly localized in mitochondria (Figure 5C, bottom panel). Very little fluorescent signal was evident outside of mitochondria. No background reactivity was obtained with the pre-immune serum (Figure 5C, top panel). The LdUfc1 antibodies specifically reacted with a ∼26 kDa protein in the wild type *Leishmania* promastigote lysates (Figure 5D, lane α-LdUfc1). The pre-immune serum did not react with any protein in the *Leishmania* lysates (Figure 5D, NRS). Immunofluorescence assay with the anti-LdUfc1 antibodies showed that endogenous *Leishmania* Ufc1 protein in promastigotes is clearly localized in mitochondria (Figure 5E, bottom panel). No fluorescent signal was observed outside of mitochondria. Control staining with the pre-immune serum only showed background reactivity (Figure 5E, top panel). Taken together the cellular distribution of LdUfm1, LdUba5, LdUfc1 and the Ufm1 conjugated substrates in the mitochondria argues for a mitochondrion specific Ufm1 conjugation system in *Leishmania*.

### LdUfm1 is conjugated to mitochondrial proteins

**Figure 6:**
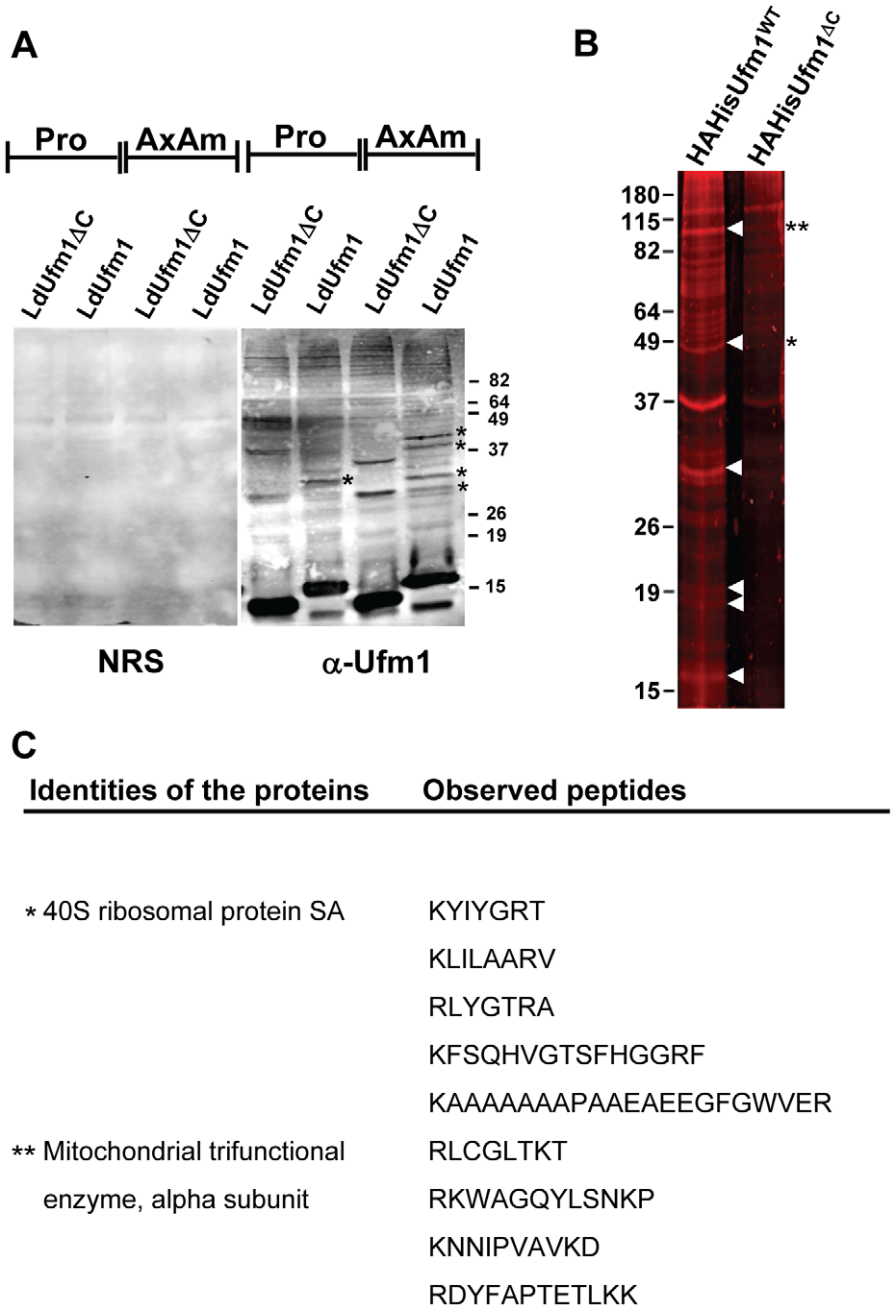
Purification of LdUfm1 conjugates and identification of the peptides by mass spectrometric analysis. (A) Protein lysates either from promastigote stage (Pro) or axenic amastigote (AxAm) stage *Leishmania* transfectant cells expressing either HA-His-Ufm1^WT^ or HA-His-Ufm^ΔC^ were prepared under denaturing conditions and the conjugates were precipitated with Ni-agarose beads. The eluates from the beads were subjected to SDS-PAGE and the immunoblots were probed with α-Ufm1 antibodies. Pre-immune serum (NRS) was used in control immunoblots. (B) *Leishmania* transfectant cells expressing HA-His-Ufm1^WT^ or the non-conjugatable HA-His-Ufm^ΔC^ were lysed under denaturing conditions and the conjugates were precipitated with Ni-agarose beads. The eluates from the beads were subjected to SDS-PAGE stained with Coomassie blue and scanned on a LiCor odyssey instrument. The SDS-gel slices excised from the acrylamide gel for the purpose of peptide identification are indicated with white arrow marks. (C) The identification of the peptides from the gel slices and the corresponding proteins to which the peptides map to are listed. The asterisks (B and C) indicate the gel slices from which the peptides originated.

The existence of LdUba5 and LdUfc1 strongly indicated that LdUfm1 mediated protein conjugation occurs in this parasite. Direct conjugation by E2 enzymes to protein targets has been shown to occur independent of E3 ubiquitin ligases [29]. Based on this reasoning, we investigated whether LdUfm1 conjugation could occur with its substrates in *Leishmania*. We constructed HA and 6xHis tag containing LdUfm1 that ends in the Gly98 residue at the C-terminus and a variant of LdUfm1 which lacks a Gly residue at the C-terminus and has instead Val97 as its terminal residue. This mutant lacking the terminal Gly therefore must be unable to conjugate to the protein targets in *Leishmania* and therefore should serve as a background control. Using these transfectants, LdUfm1 conjugates were purified using Nickel agarose beads that bound the 6xHis tag in the transfected LdUfm1. This purification was carried out under denaturing conditions to avoid purifying proteins that interact non-covalently with LdUfm1. Results showed that distinct Ufm1 conjugates from promastigotes and axenic amastigotes were identified by α-Ufm1 antibody (Figure 6A) suggesting the subtle variations in the Ufm1 mediated conjugations that might accompany the parasitic differentiation. Using similar protocol, we purified the Ufm1 conjugated for the purpose of identification by mass spectrometric analysis. Results showed that several proteins with molecular weights ranging from ∼20 to ∼100 kDa were detected only in the transfectants expressing mature LdUfm1 but not in the transfectants expressing the non-conjugatable LdUfm1 (Figure 6B). The SDS-PAGE gel slices corresponding to these conjugates were excised and in-gel tryptic digestion was performed followed by reduction and alkylation reactions to generate peptides. The peptides were analyzed by LC/MS and the product ion spectra were searched against the non-redundant NCBI database and *Leishmania infantum* GeneDB database (http://www.genedb.org/genedb/leish/) using the Mascot search program. Out of the multiple *Leishmania* proteins to which the tryptic peptides mapped only those proteins where multiple peptides could be assigned are shown (Figure 6C). The peptides in each protein identified came from gel slices that had higher calculated molecular weight than the predicted molecular weight of the identified protein suggesting post translational modifications, possibly with LdUfm1. Our LC/MS data identified two proteins 40S ribosomal protein SA (from the gel slice corresponding to ∼50 kDa) and mitochondrial trifunctional protein α-subunit (from the gel slice corresponding to ∼95 kDa) with multiple peptides mapped to the corresponding proteins. Interestingly, both these proteins have been shown to have mitochondrial localization in trypanosomatids [30], [31]. Taken together, these data demonstrate that for the first time a unicellular organism such as the *Leishmania* parasites possesses the full complement of Ufm1 mediated protein conjugation machinery which seems to be functioning in the mitochondria.

## Discussion

In this report we demonstrated the existence of an Ufm1 conjugation pathway in the human protozoan parasite *Leishmania donovani* including the enzymatic steps involving activation by Uba5 (E1), conjugation by Ufc1 (E2) and finally conjugation with substrate proteins. Modification and/or alterations in the expression of Ufm1 and Uba5 showed parasite stage specific growth effects underlining the importance of Ufm1 mediated protein modifications in *Leishmania* growth and differentiation. Importantly all the components of Ufm1 conjugation and the substrate proteins in *Leishmania* appear to be associated with the mitochondria. To our knowledge this is the first demonstration of a trypanosomatid mitochondrial associated complete Ubl pathway in which the activation, conjugation components and the substrate proteins are all localized in mitochondria.

Initial studies showed that the Ufm1 pathway is widely conserved in mammals, nematode and other multicellular organisms but absent in unicellular eukaryotes such as yeast [24]. Our first of a kind demonstration of Ufm1 pathways in trypanosomatid parasites, unicellular eukaryotes that emerged as early eukaryotic organisms indicates the ancient origins of this pathway. In trypanosomatid parasites, our analysis showed that the Ubls such as Ub, SUMO and Ufm1 appear to have one or more aminoacid residues after the C-terminal glycine that need to be processed before conjugation (data not shown). Among the trypanosomatids, only *Leishmania* Ufm1 has a longer C-terminal extension that is 17 residues long. However, our results suggest that in *L. donovani* C-terminal processing of Ufm1 does occur suggesting the presence of a processing protease and the Gly98 residue is essential for that cleavage and subsequent conjugation. In contrast, apicomplexan parasites such as *Plasmodium* contain Ubls (Atg8) that terminate in a C-terminal Glycine indicating the absence of processing prior to conjugation [23]. This structural diversity among different parasites might be a result of adaptations that parasites have undergone in their respective environments during their complex life cycles.

Similar to humans, LdUba5 interacts with LdUfm1 and activates it in an ATP dependent reaction. Also, biochemical characteristics shared between human Uba5 and LdUba5 and activation of human Ufm1 by LdUba5 demonstrated the mechanistic similarities between the pathways in the two species. Previously it has been demonstrated that human Ufm1 interacts with its activating enzyme Uba5 involving the cysteine residue in the active site [24]. Also, it has been shown in many of the E1 and E1-like enzymes of Ub or Ubls that changing the cysteine residue in the active site to serine results in formation of stable intermediates between the E1 and its respective modifier protein. Our studies showed that LdUba5^C217S^ mutant also makes a stable intermediate with LdUfm1. The formation of stable intermediate is likely due to the covalent ester link between Uba5 and Ufm1 as previously observed in other E1-like enzymes [24]. It is reasonable to expect overexpression of LdUba5^C217S^ would have a negative dominant effect which will result in depletion of the free pool of endogenous Ufm1. Interestingly, overexpression of the LdUba5^C217S^ mutant resulted in significant reduction of *L. donovani* amastigote growth suggesting a role of LdUfm1 conjugation in parasite pathogenesis (Figure 4B). Similarly, protein interactions between LdUfm1 and LdUfc1 showed the conservation of E2-like activity in *L. donovani* parasites, thereby demonstrating the importance of E2 activity in the *Leishmania* Ufm1 conjugation pathway as well as E1 activity. Recently, human E3-ligase Ufl1 that conjugates Ufm1 to its substrate protein was identified [26]. Database searches in the trypanosomatid genomes indicated that these parasites may encode an ortholog of human ufl1, albeit with low sequence homology. However, E3 ligase independent conjugation of Ubls via E2 activity is still a distinct possibility as was recently reported [29].

One of the primary reasons for the inability to assign any biological function to the Ufm1 pathway in any organism so far is that the target proteins that are conjugated by Ufm1 have not been characterized. Our LC/MS results showed that LdUfm1 is potentially conjugated to the 40S ribosomal protein SA and the mitochondrial trifunctional protein α-subunit. It is known that the 40S ribosomal protein SA is required for the assembly and/or stability of the 40S ribosomal subunit. In *L. tarentolae* 40S ribosomal subunits have been shown to be enriched in mitochondria [30]. A role for Ubls in the regulation of either stability or activity of mammalian ribosomal proteins was indicated by their conjugation to NEDD [32].

The mitochondrial trifunctional protein catalyzes three consecutive reactions in the β-oxidation of long-chain fatty acids, and plays important role in control and regulation of β-oxidation [33]. Fatty acid oxidation is an essential energy generation system that occurs in mitochondria. In *T. brucei*, a trypanosomatid parasite related to *Leishmania*, proteomic analysis revealed that the mitochondrial trifunctional protein is enriched in the mitochondria [31]. In trypanosomatids the regulation of β-oxidation of long chain fatty acids is not completely understood. Further studies should reveal if conjugation with Ufm1 has any role in altering the activity or signaling the translocation of the mitochondrial trifunctional protein during this energy generation process. Recently, a protein with a suggested role in membrane transport was reported as the first identified target of human Ufm1 modification [26].

Our results showed that in *L. donovani* Ufm1, Uba5, and Ufc1 are found associated with the mitochondrion. In addition, both the targets for Ufm1 conjugation that we identified in this study also are associated with the mitochondrion. The presence of the components of *Leishmania* Ufm1 pathway and its substrates argue for the existence of a mitochondria associated Ufm1 conjugation pathway. In contrast, subcellular localization of human Ufm1as well as other components i.e., Uba5, Ufc1 or Ufl1 is not known [24], [26]. Previously E1, E2 of Ub or E3 of SUMO were shown independently to be associated with mitochondria in metazoans [34]–[36]. To our knowledge, this is the first report that demonstrates the presence of complete Ufm1 pathway along with its substrates in the mitochondria.

Mitochondrion associated Ufm1 conjugation in *Leishmania* presents several interesting possibilities regarding biological functions of this pathway. For example, ubiquitin involvement in the degradation of mitochondrial matrix proteins is known [37]. Stability of several mammalian mitochondrial proteins is affected by proteasomal inhibition suggesting a role for ubiquitination [38]. Several E3 ligases located on the outer mitochondrial membrane have been identified recently that primarily affect the mitochondrial morphology by either ubiquitination [39] or by Sumoylation of mammalian proteins that regulate mitochondrial fission [35]. It would be of interest to investigate whether the changes mediated by the Ufm1 conjugation have any impact on the life-cycle related differentiation of the parasites.

Demonstration of the existence of mitochondria associated Ufm1 conjugation in unicellular trypanosomatid parasites offers unique possibilities to explore the importance of this pathway in the context of its pathogenesis and host-parasite adaptations. Importantly, the ubiquitin-dependent proteolysis system (UPS) is increasingly recognized as a viable therapeutic pathway in the treatment of cancer after the successful treatment of hematological malignancies with proteasome inhibitors [40]. Deubiquitinases, the key effectors of UPS and intracellular signaling cascades, and Ub ligases because of their narrow substrate specificity are emerging as important targets for potential anticancer therapies [41], [42]. Identification of Ufm1 mediated protein modification pathways in *Leishmania*, with its distinct subset of substrate proteins associated with mitochondrial activities as demonstrated in this report may provide specific targets for novel drug therapies against this human pathogen.

## Materials and Methods

### Plasmids and parasite cultures

*Leishmania donovani* promastigotes (strain 1S, clone 2D, WHO designation: MHOM/SD/62/1S-CL2D) were grown in M199 medium containing 10% heat inactivated fetal bovine serum. Promastigotes were transfected by electroporation and selected for growth in medium containing Geneticin (G418) up to 100 µg/ml or Hygromycin up to 50 µg/ml or both. These drug-resistant cells were used in all subsequent experiments. The *Leishmania* expression plasmid pKSNeo [43] was used to express either the full-length or where indicated, the mutant forms of *L. donovani* Ufm1, Uba5 or Ufc1 in transfected *Leishmania* parasites. For this purpose, the full-length gene encoding *L. donovani* Ufm1 was amplified with oligos based on the *L. infantum* putative Ufm1 sequence. The point mutation G98A was introduced by PCR to generate Ufm1 mutants. These oligos introduced a FLAG tag at the C' terminus of the fusion protein and contained *Spe*I restriction sites on either end. The *Spe*I insert was subcloned into the *Spe*I site of pKSNeo, resulting in plasmid constructs that were used to overexpress wild type or mutant Ufm1 in *Leishmania* transfectants. For co-expression experiments pXGHYG plasmid was used [44].

### Bacterial expression of LdUba5, LdUfc1 and TbUfm1

Wild type LdUba5, LdUfc1 and TbUfm1 were amplified by PCR and ligated into pCR T7/CT-Topo (Invitrogen). The recombinant proteins were expressed from *E. coli* and purified in native conditions through Ni-agarose column chromatography according to the manufacturer's protocol (Qiagen). The recombinant purified LdUba5, LdUfc1 and TbUfm1 proteins were used to generate polyclonal antibodies in rabbits according to the manufacturer's protocol (Spring Valley Laboratories, Woodbine, MD).

### Ufm1 activation by LdUba5

Human recombinant Ufm1 and Uba5 were purchased from Boston Biochem, Cambridge, MA. Leishmania Uba5, Ufm1 wild type and mutant Uba5 and Ufm1 proteins were immunoprecipitated from the respective transfectants using anti-HA affinity gel. These immunoprecipitated proteins were used in combinations in a fluorescent polarization assay (Transcreener AMP/GMP Assay) using reagents purchased from Bell Brook labs, Madison, WI and FP measurements were obtained from Spectramax M5 fluorescence spectrometer. The Transcreener AMP/GMP Assay allows measurement of any enzymatic activity that produces AMP. The fluorescent signal is emitted by an AMP/GMP Alexa633 tracer bound to an AMP/GMP Antibody. When the tracer is displaced by the AMP generated during E1-like enzymatic activity, it results in free rotation of the tracer leading to a decrease in polarization. The amount of AMP released in the LdUfm1 activation is proportional to the decrease in polarization.

### Identification of conjugates of LdUfm1 by mass spectrometric analysis

For immunoprecipitation analysis, 1×10^8^ *Leishmania* promastigotes were lysed by 1 ml of NET buffer (150 mM NaCl, 1 mM EDTA, 10 mM Tris–HCl, pH 7.5, 1% Nonidet P-40 with protease inhibitor cocktail) and the lysate was centrifuged at 12000 rpm for 20 min at 4°C to collect the supernatant. To 500 µl of the lysate, 5 µl of anti-Flag-M2 antibodies (Sigma) were added to the lysate and the mixture was incubated under constant rotation over night at 4°C. The complexes were immunoprecipitated with 25 µl of Protein-A Sepharose beads by incubation under constant rotation at 4°C for 1 hr. The precipitated complexes were washed five times with ice-cold NET buffer and eluted by boiling for 5 min in SDS sample buffer in the presence of β-mercaptoethanol. The supernatant was subjected to SDS–PAGE and analyzed by immunoblots with anti-Flag (M2) or anti-HA antibody.

For purification of 6xHis-tagged proteins under denaturing conditions, 2×10^8^ transfectant *Leishmania* promastigotes or axenic amastigotes were lysed by 1 ml of denaturing lysis buffer (8M urea, 0.1M NaH_2_PO_4_, and 0.01M Tris-HCl, pH 8.0 with 20 mM N-ethylmaleimide) and the lysate was sonicated briefly and then centrifuged at 12000 rpm for 20 min at room temperature to remove debris. To the supernatant, 30 µl of Ni-NTA Agarose (Qiagen) was added and the mixture was incubated under constant rotation for 30 min at room temperature. The precipitated material was washed five times with denaturing wash buffer (8M urea, 0.1M NaH_2_PO_4_, and 0.01M Tris-HCl, pH 5.9). The complexes were eluted in elution buffer (8M urea, 0.1M NaH_2_PO_4_, and 0.01M Tris-HCl, pH 4.5). The resulting lysates were subjected to SDS-PAGE and analyzed by immunoblots with α-TbUfm1.

### Intracellular localization of LdUfm1, LdUba5, LdUfc1

Immunofluorescence assay was performed essentially as described previously (Gannavaram et al., 2008). Briefly, the cells were preincubated with Mitotracker Red (Invitrogen) to label the mitochondria. Rabbit polyclonal antibodies raised against bacterially expressed TbUfm1, LdUba5 or LdUfc1 proteins were used to detect LdUfm1, LdUba5, LdUfc1 in *Leishmania* promastigotes to determine the cellular localization of endogenous proteins in these cells.

### Macrophage infection

Human elutriated monocytes were resuspended at 1.8×10^5^ cells/ml in RPMI medium containing 10% FBS macrophage colony-stimulating factor (20 ng/ml, ProSpec, Israel), plated in 0.5 ml on eight-chamber Lab-Tek tissue-culture slides (Miles Laboratories) and incubated for 9 days for differentiation into macrophages. The macrophage infection experiments were performed essentially as described earlier [28].

## Supporting Information

Figure S1 **Trypanosomatid parasite genomes contain homologs of mammalian Ufm1.** CLUSTAL W alignment of *Leishmania donovani, Trypanosoma brucei* and *Trypanosoma cruzi* putative Ufm1 with the mammalian homolog. The shaded area represents identical residues, and dashes represent gaps. The C'terminal glycine, essential residue for the substrate conjugation is indicated with arrow mark. The percentage identities of full-length *L. donovani* Ufm1 with those of *T. brucei*, *T. cruzi* and human Ufm1 are shown in the parentheses.
(TIF)

Figure S2 **Trypanosomatid parasite genomes contain homologs of mammalian Uba5.** Sequence alignment of *Leishmania donovani, Trypanosoma brucei* and *Trypanosoma cruzi* putative Uba5 with the mammalian homologs. The shaded area represents identical residues, and dashes represent gaps. The ATP-binding motif involving the residues GXGXXG is indicated with a solid line. The putative active site Cys residue is indicated with an asterisk. The metal-binding motif is indicated with a dotted line. The percentage identities of full-length *L. donovani* Uba5 with those of *T. brucei*, *T. cruzi* and human Uba5 are shown in the parentheses.
(TIF)

Figure S3 **Trypanosomatid parasite genomes contain homologs of mammalian Ufc1.** CLUSTAL W alignment of *Leishmania donovani, Trypanosoma brucei* and *Trypanosoma cruzi* putative Ufc1 with the mammalian homolog. The shaded area represents identical residues, and dashes represent gaps. The putative active site Cys residue is indicated with an asterisk. The percentage identities of full-length *L. donovani* Ufm1 with those of *T. brucei*, *T. cruzi* and human Ufc1 are shown in the parentheses.
(TIF)

Figure S4 **Purification of LdUfm1 conjugates from LdUfm1 transfectant amastigotes.** (A) *Leishmania* transfectant cells expressing HA-6xHis-Ufm1 or HA-6xHis-Ufm1^G98A^ or the non-conjugatable HA-6xHis-Ufm^ΔC^ were lysed under denaturing conditions and the conjugates were precipitated with Ni-agarose beads. The eluates from the beads were subjected to SDS-PAGE and the immunoblots were probed with anti-Ufm1 antibodies and scanned on a LiCor odyssey instrument. The conjugates are indicated with asterisk and the unconjugated Ufm1 is indicated with arrows.
(TIF)
