# Supplementary figures and images for: Mitochondrial Associated Ubiquitin Fold Modifier-1 Mediated Protein Conjugation in Leishmania donovani
Source: PLoS One. 2011 Jan 14;6(1):e16156. doi: 10.1371/journal.pone.0016156 (PMC3021533; doi:10.1371/journal.pone.0016156)

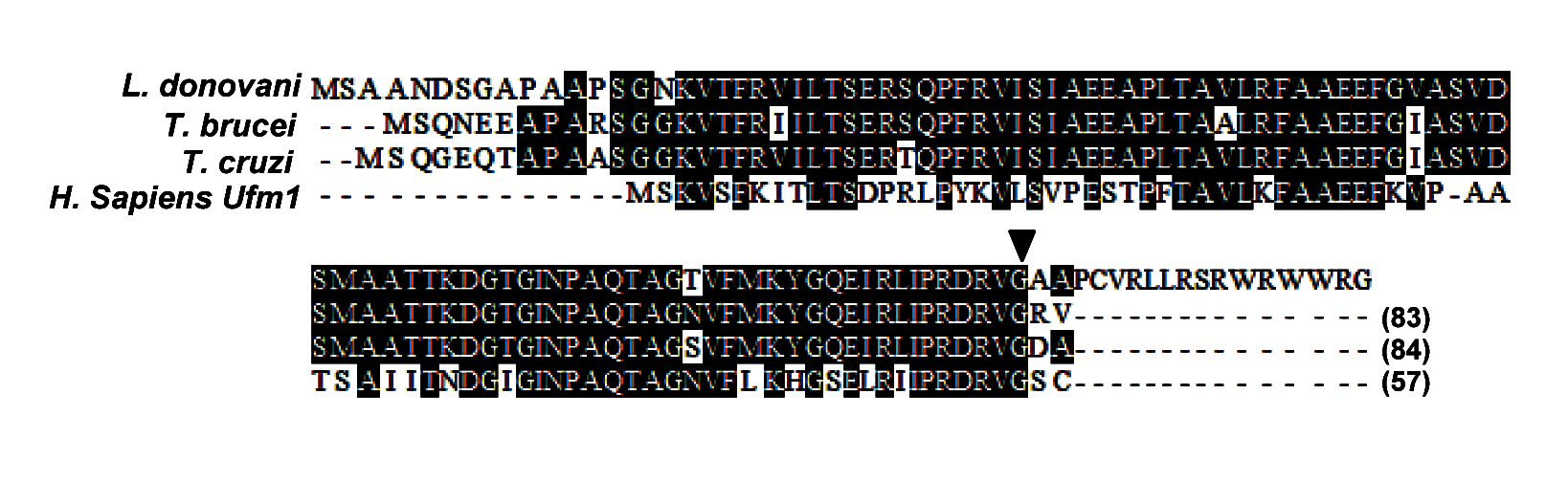

Supplement: Figure S1 — Trypanosomatid parasite genomes contain homologs of mammalian Ufm1. CLUSTAL W alignment of Leishmania donovani, Trypanosoma brucei and Trypanosoma cruzi putative Ufm1 with the mammalian homolog. The shaded area represents identical residues, and dashes represent gaps. The C'terminal glycine, essential residue for the substrate conjugation is indicated with arrow mark. The percentage identities of full-length L. donovani Ufm1 with those of T. brucei, T. cruzi and human Ufm1 are shown in the parentheses. (TIF) [file pone.0016156.s001.tif]

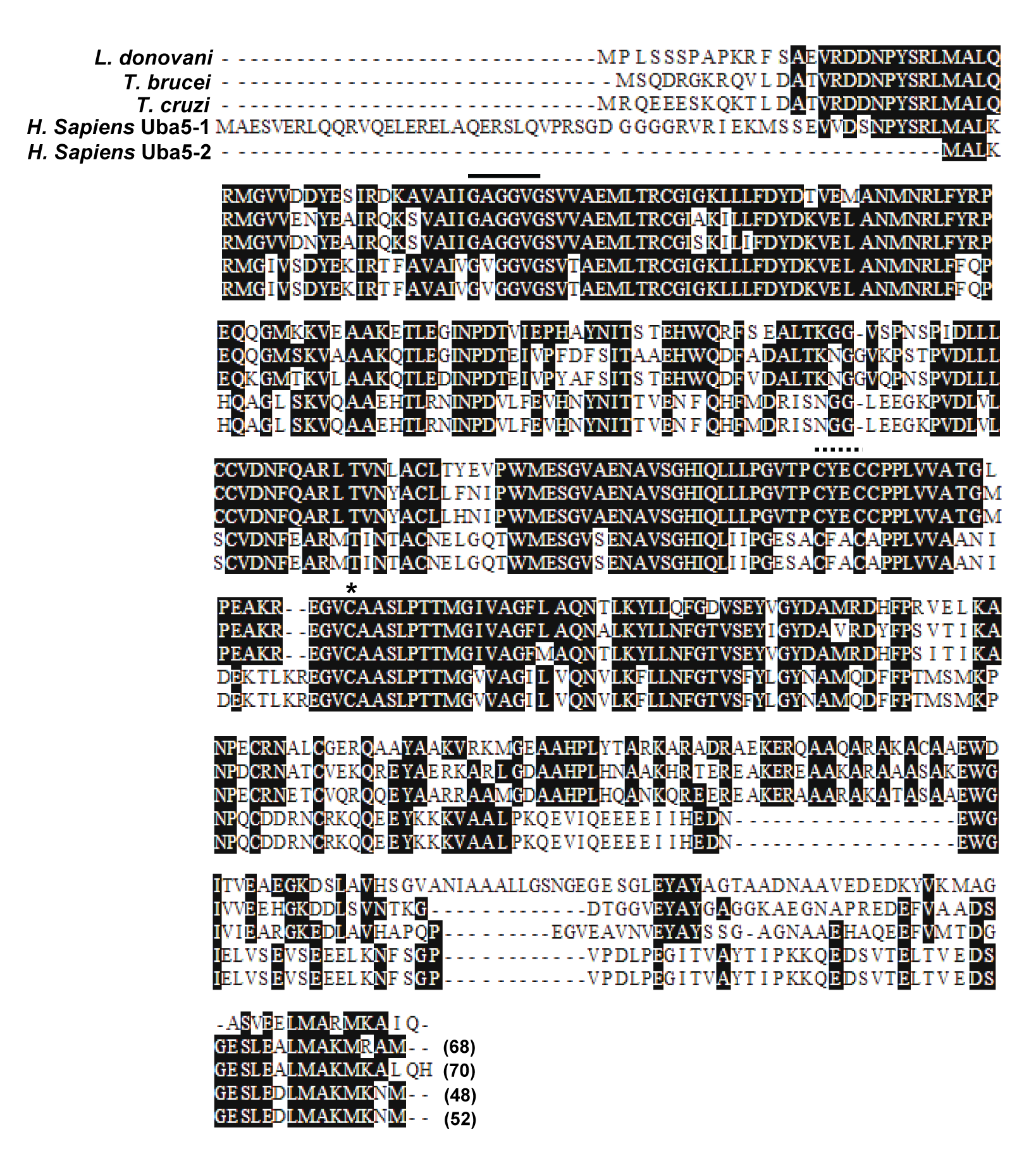

Supplement: Figure S2 — Trypanosomatid parasite genomes contain homologs of mammalian Uba5. Sequence alignment of Leishmania donovani, Trypanosoma brucei and Trypanosoma cruzi putative Uba5 with the mammalian homologs. The shaded area represents identical residues, and dashes represent gaps. The ATP-binding motif involving the residues GXGXXG is indicated with a solid line. The putative active site Cys residue is indicated with an asterisk. The metal-binding motif is indicated with a dotted line. The percentage identities of full-length L. donovani Uba5 with those of T. brucei, T. cruzi and human Uba5 are shown in the parentheses. (TIF) [file pone.0016156.s002.tif]

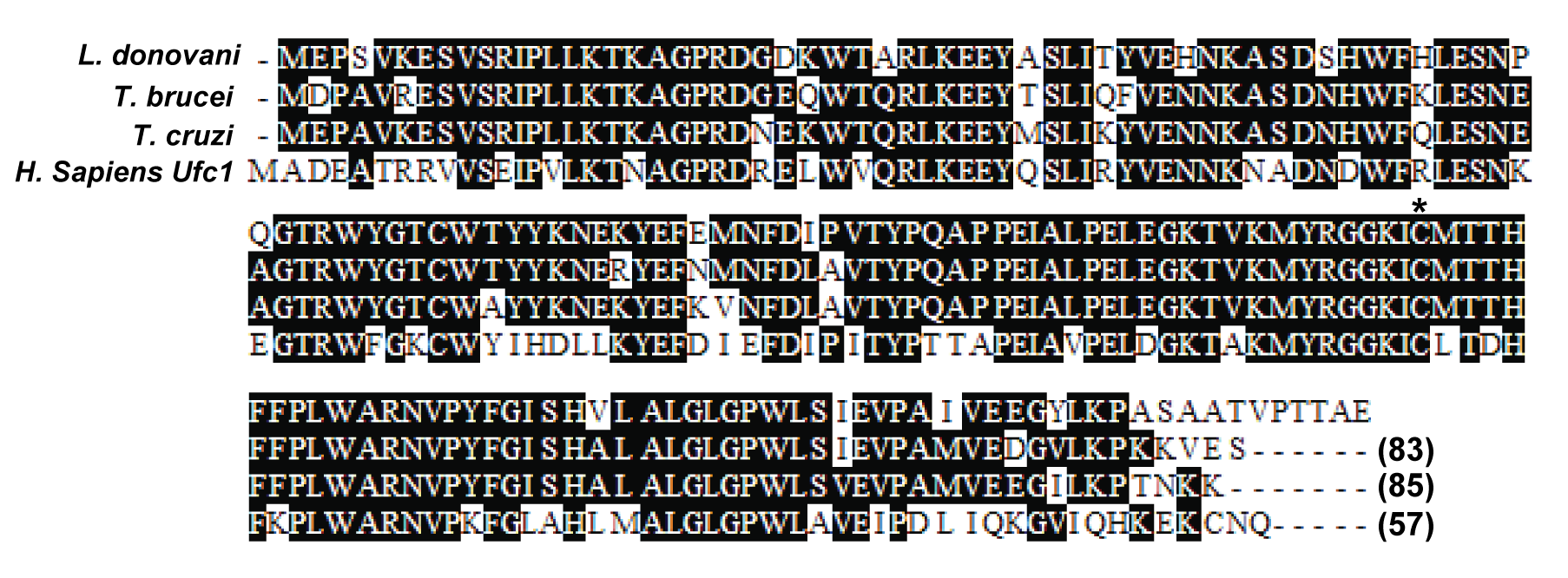

Supplement: Figure S3 — Trypanosomatid parasite genomes contain homologs of mammalian Ufc1. CLUSTAL W alignment of Leishmania donovani, Trypanosoma brucei and Trypanosoma cruzi putative Ufc1 with the mammalian homolog. The shaded area represents identical residues, and dashes represent gaps. The putative active site Cys residue is indicated with an asterisk. The percentage identities of full-length L. donovani Ufm1 with those of T. brucei, T. cruzi and human Ufc1 are shown in the parentheses. (TIF) [file pone.0016156.s003.tif]

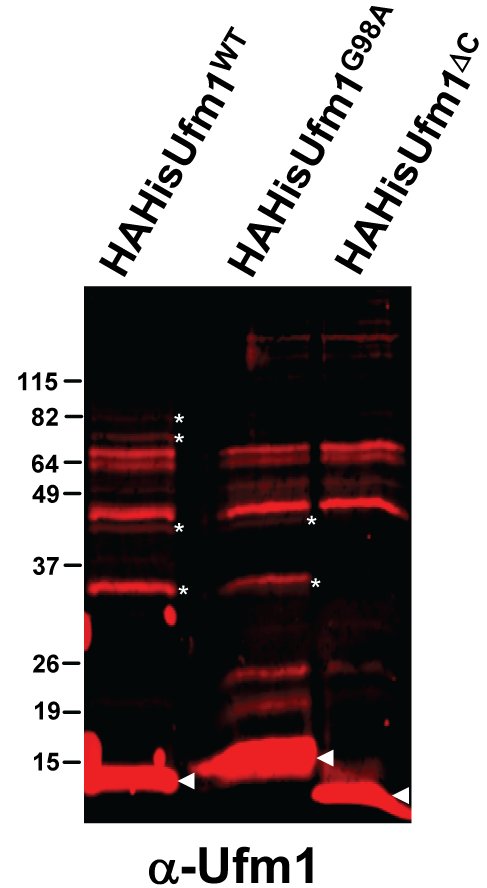

Supplement: Figure S4 — Purification of LdUfm1 conjugates from LdUfm1 transfectant amastigotes. (A) Leishmania transfectant cells expressing HA-6xHis-Ufm1 or HA-6xHis-Ufm1G98A or the non-conjugatable HA-6xHis-UfmΔC were lysed under denaturing conditions and the conjugates were precipitated with Ni-agarose beads. The eluates from the beads were subjected to SDS-PAGE and the immunoblots were probed with anti-Ufm1 antibodies and scanned on a LiCor odyssey instrument. The conjugates are indicated with asterisk and the unconjugated Ufm1 is indicated with arrows. (TIF) [file pone.0016156.s004.tif]
